# Heritability of hypothyroidism in the Finnish Hovawart population

**DOI:** 10.1186/s13028-016-0221-8

**Published:** 2016-06-07

**Authors:** Johanna Åhlgren, Pekka Uimari

**Affiliations:** Department of Agricultural Sciences, University of Helsinki, Helsinki, Finland

**Keywords:** Dog, Gibbs sampling, Heritability, Hovawart, Hypothyroidism, Probit model

## Abstract

**Background:**

The Hovawart is a working and companion dog breed of German origin. A few hundred Hovawart dogs are registered annually in Finland. The most common disease with a proposed genetic background in Hovawarts is hypothyroidism. The disease is usually caused by lymphocytic thyroiditis, an autoimmune disorder which destroys the thyroid gland. Hypothyroidism can be treated medically with hormone replacement. Its overall incidence could also be reduced through selection, provided that the trait shows an adequate genetic basis. The aim of this study was to estimate the heritability of hypothyroidism in the Finnish Hovawart population.

**Results:**

The pedigree data for the study were provided by the Finnish Kennel Club and the hypothyroidism data by the Finnish Hovawart Club. The data included 4953 dogs born between 1990 and 2010, of which 107 had hypothyroidism and 4846 were unaffected. Prior to the estimation of heritability, we studied the effects of gender, birth year, birth month, and inbreeding on susceptibility to hypothyroidism. Heritability was estimated with the probit model both via restricted maximum likelihood (REML) and Gibbs sampling, using litter and sire of the dog as random effects. None of the studied systematic effects or level of inbreeding had a significant effect on susceptibility to hypothyroidism. The estimated heritability of hypothyroidism varied from 0.47 (SE = 0.18) using REML to 0.62 (SD = 0.21) using Gibbs sampling.

**Conclusions:**

Based on our analysis, the heritability of hypothyroidism is moderate to high, suggesting that its prevalence could be decreased through selection. Thus, breeders should notify the breed association of any affected dogs, and their use for breeding should be avoided.

## Background

Hypothyroidism is one of the most common hereditary diseases in Finnish Hovawart dogs, and actually the most common endocrine disease in all dogs [1]. The prevalence of hypothyroidism between different breeds varies from 0.2 to 0.9 % [2–4]. In some breeds considerable higher prevalence have been reported e.g. 2.7 % for a cohort of 8 year old Gordon setters [5], 13 % for Swedish Hovawarts [6], and as high as 16 % for Giant Schnauzers [6] and for Beagles [7]. The disease is rarely diagnosed in dogs under the ages of 2–4 years [8]. Hypothyroidism is mainly caused by lymphocytic thyroiditis or idiopathic thyroid atrophy [6]. Lymphocytic thyroiditis is considered as an autoimmune disorder [9, 10] and has a hereditary predisposition [2, 7, 11]. It also has been suggested that the disease may be influenced by a major gene [11]. Lymphocytic thyroiditis is known to be associated with the major histocompatibility complex (MHC) or dog leukocyte antigen (DLA) system class II allele [5, 12–14]. Several DLA haplotypes which increase the risk to acquire hypothyroidism have been identified in different breeds [5, 12, 14]. However, the recent whole genome association study with SNP (single nucleotide polymorphism) markers indicated that a separate region in the vicinity of DLA is also associated with hypothyroidism in dogs [15]. The association between certain DLA haplotypes and the risk of hypothyroidism together with the immune-mediated nature of this disease [16, 17] leads to the hypothesis that inbreeding increases its incidence. Gender and neutering may also raise the incidence of hypothyroidism: a higher incidence of hypothyroidism has been observed for castrated males and females than for uncastrated males and females [2, 3] and a higher incidence for females than for males [3]. However, the genetic background of hypothyroidism has not been studied extensively, and only a few heritability estimates have been reported. Benjamin et al. [7] obtained a moderate estimate of heritability (0.3) based on 276 laboratory Beagle dogs with 16 % diagnosed as hypothyroid, and a slightly smaller estimate (0.2) based on over 1000 laboratory Beagles in an experiment designed to study the effect of radiation on the risk of hypothyroidism [11]. The heredity estimates presented here for hypothyroidism in the Finnish Hovawart population are based on a pedigree analysis of 107 dogs affected with hypothyroidism and 4846 unaffected dogs. We also tested the effects of gender, birth year, birth month, and level of inbreeding on the dogs’ susceptibility to hypothyroidism.

## Methods

**Fig. 1:**
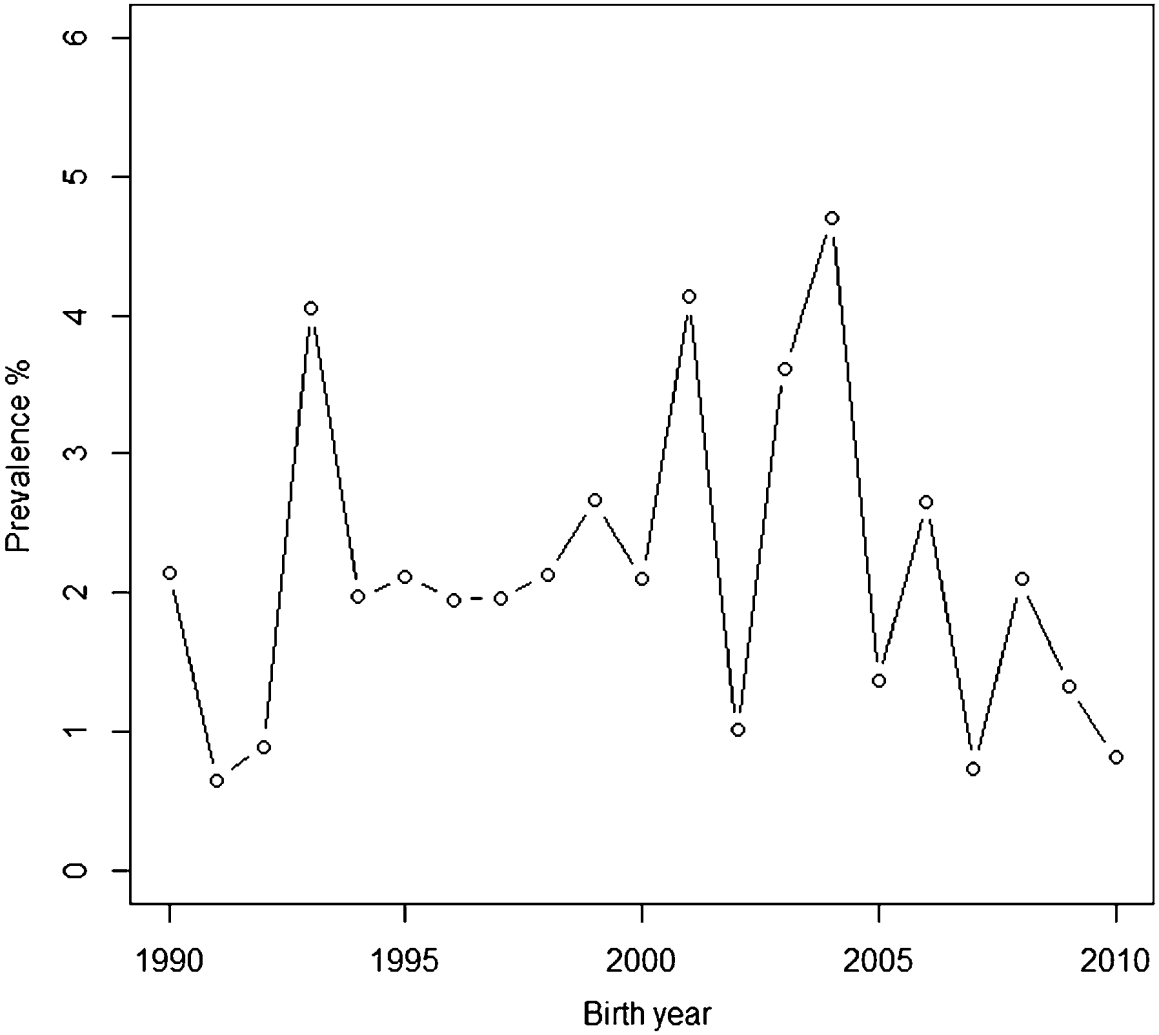
Prevalence of hypothyroidism in Finnish Hovawart dogs born between 1990 and 2010

The list of dogs diagnosed with hypothyroidism was provided by the Finnish Hovawart Club. Only dogs born between 1990 and 2010 were used in this study resulting to 107 affected dogs in the data. The prevalence of hypothyroidism in Finnish Hovawart dogs by birth year is presented in Fig. 1. The dogs with hypothyroidism were self-reported by the breeders or the owners of the dogs to the breed association and were based on diagnoses made by different veterinarians using clinical signs and laboratory tests [see e.g. 18]. The upper birth year of 2010 was selected so that the youngest dogs were at least 2–4 years-of-age at the time the affection status data were received from the breed association in September 2014. Some of these dogs may have developed hypothyroidism at older age after that. The pedigree data were obtained from the Finnish Kennel Club and included 7178 animals. Dogs not included in the list of dogs with hypothyroidism and born between 1990 and 2010 were extracted from the pedigree file, a total of 4846 dogs, and treated as unaffected. Based on the phenotypic data, the prevalence of hypothyroidism was 2.2 %. Eight sires had two affected progeny, three sires had three affected progeny, four sires had four affected progeny, and one sire had seven affected progeny in multiple litters. Of the dams, seven had two affected progeny, four dams had three affected progeny, three dams had four affected progeny, and one dam had five affected progeny.

Prior to genetic analysis, we studied the influence of gender, birth year, birth month, and level of inbreeding on hypothyroidism susceptibility using the generalized linear model in IBM SPSS Statistics v.21. Birth months were classified into four seasons: December to February, March to May, June to August, and September to November. The effect of inbreeding on susceptibility to hypothyroidism was tested by logistic regression, only including individuals born between 1990 and 2010 and having a pedigree completeness value [19] of over 90 % based on five ancestral generations. This criterion was met by 4115 dogs.

Variance components were estimated via the restricted maximum likelihood (REML) method [20] with a probit link function assuming an underlying, unobservable, normally distributed liability [21]. The following linear model was used for the variance component estimation:

$${\text{probit }}( {{\text{p}}_{\text{ijk}} } ) = { \upmu } + {\text{ birthy}}_{\text{i}} + {\text{ litter}}_{\text{j}} + {\text{ sire}}_{\text{k}} ,$$

where ${\text{probit}}( {{\text{p}}_{\text{ijk}} } ) \, = \Upphi^{ - 1} \times P( {{\text{y}}_{\text{ijk}} = { 1}} ).\,P( {{\text{y}}_{\text{ijk}} = { 1}} )$ is the probability that the dog has hypothyroidism given that its sire is k and it was born in litter j during year (birthy) i. Φ is the cumulative density function of the underlying normal distribution. The random sire effect was assumed to have a normal distribution with mean 0 and a variance–covariance structure of ${\mathbf{A}}{\upsigma }_{\text{s}}^{2}$, where **A** is the additive relationship matrix between the sires and maternal grandsires and ${\upsigma }_{\text{s}}^{2}$ is the sire variance. The litter effect was also a random effect with a normal distribution with mean 0 and a variance structure of ${\mathbf{I}}{\upsigma }_{\rm l}^{2}$, where **I** is the identity matrix with diagonal elements of ${\upsigma }_{\rm l}^{2}$ (litter variance). Residual variance (${\upsigma }_{\text{e}}^{2}$) was fixed at 1. The heritability of the underlying liability was estimated as:

1 $$\text{h}^{2} { = }\frac{{4\upsigma_{\text{s }}^{2} }}{{ \upsigma_{\text{l}}^{2} + \upsigma_{\text{s}}^{2} { + 1}}}$$

Additionally, we estimated the variance components using a Bayesian analysis via Gibbs sampling. The theoretical basis of the Gibbs sampling approach for mixed linear models is presented by Wang et al. [22] and is not repeated here. The length of the Gibbs chain was set to 205,000, where the first 5000 realizations of the parameter estimates were discarded (burn-in period). The final posterior mean, standard deviation, and distribution were based on every 50th iteration of the chain (thinning). An uninformative prior was used for the birth year effect, and a scaled inverted Chi square distribution with v = 6 as a degree of belief parameter and variance component estimates from the REML method as a prior values for the variance components ${\upsigma }_{\rm l}^{2}$ and ${\upsigma }_{\text{s}}^{2}$, respectively [22]. A probit link function was used to link the observed binary trait (hypothyroidism status) to the unobserved normally distributed liability. Residual variance (${\upsigma }_{\text{e}}^{2}$) was fixed at 1, as with the generalized linear mixed model approach. The variance components were estimated using the DMU program package [23].

## Results and discussion

The prevalence of hypothyroidism in the Finnish Hovawart population was similar than observed previously for other breeds [2–5]. However, because the affection status was based on self-reporting by the dog owners we can expect that the true prevalence of hypothyroidism in Finnish Hovawart population is in fact higher than reported here. To obtain reliable estimates of the prevalence, the diagnostic criteria for hypothyroidism should remain the same throughout the years. In addition, a specific cohort or cross-sectional study where all the dogs fulfilling e.g. a minimum age of 4 years are diagnosed for hypothyroidism and followed-up later in their life would have also improved the quality of the data. This view is supported by the Swedish study by Ferm et al. [6] where the proportion of affected dogs increased from 6 to 13 % after all dogs that had no clinical signs nor were medically treated for hypothyroidism were tested for TgAA and THS-levels.

With our data, the gender of the dog had no effect on its susceptibility to hypothyroidism (*P* value = 0.78); the incidence of hypothyroidism in males and females was 2.1 and 2.2 %, respectively. This is contrary to the findings of Panciera [3] but in line with the results of Dixon [4] and Benjamin [7]. Neutering status was not available in our data and was not considered here. The dog’s birth year was significant with a 10 % error level (*P* value = 0.08) and was included in the final mixed model, whereas birth season was not significantly associated with hypothyroidism (*P* value = 0.21).

**Fig. 2:**
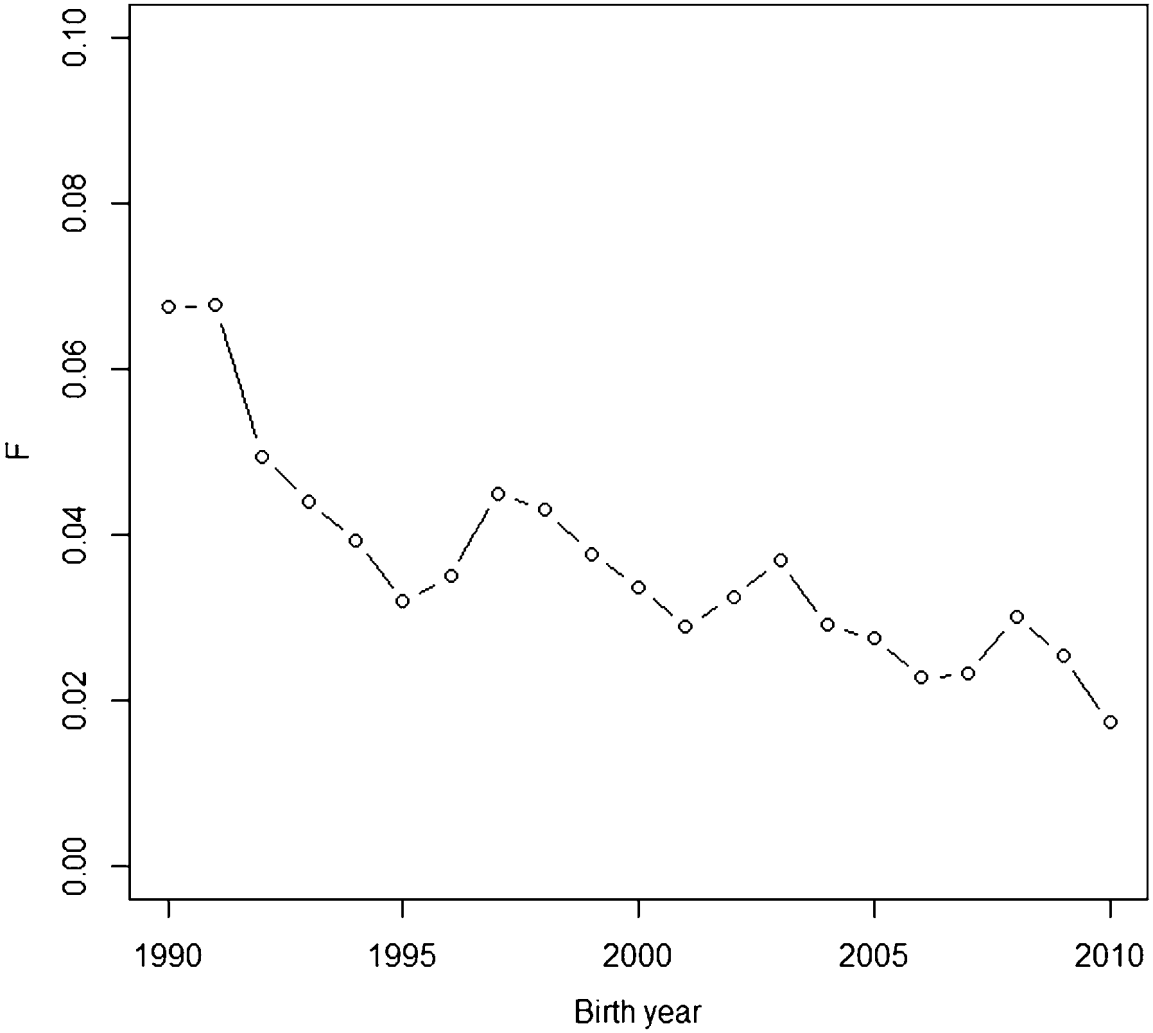
Average inbreeding coefficients of Finnish Hovawart dogs born between 1990 and 2010

The average inbreeding of Hovawart dogs born between 1990 and 2010 is presented in Fig. 2. The average level of inbreeding has generally been on the decline during the last two decades, decreasing from 6.7 % in dogs born in 1990 to less than 2 % in dogs born in 2010. Dogs diagnosed with hypothyroidism had an average inbreeding level of 3.2 %, which is lower than for dogs treated as healthy in this study (3.5 %). The regression coefficient of inbreeding from the logistic regression model was not significant (b = 1.04; SE = 2.08); thus, there was no indication that inbreeding increases the dogs’ susceptibility to hypothyroidism, at least with the inbreeding levels typical of the Finnish Hovawart population. Benjamin et al. [11] also found no effect of inbreeding on susceptibility to hypothyroidism.

**Fig. 3:**
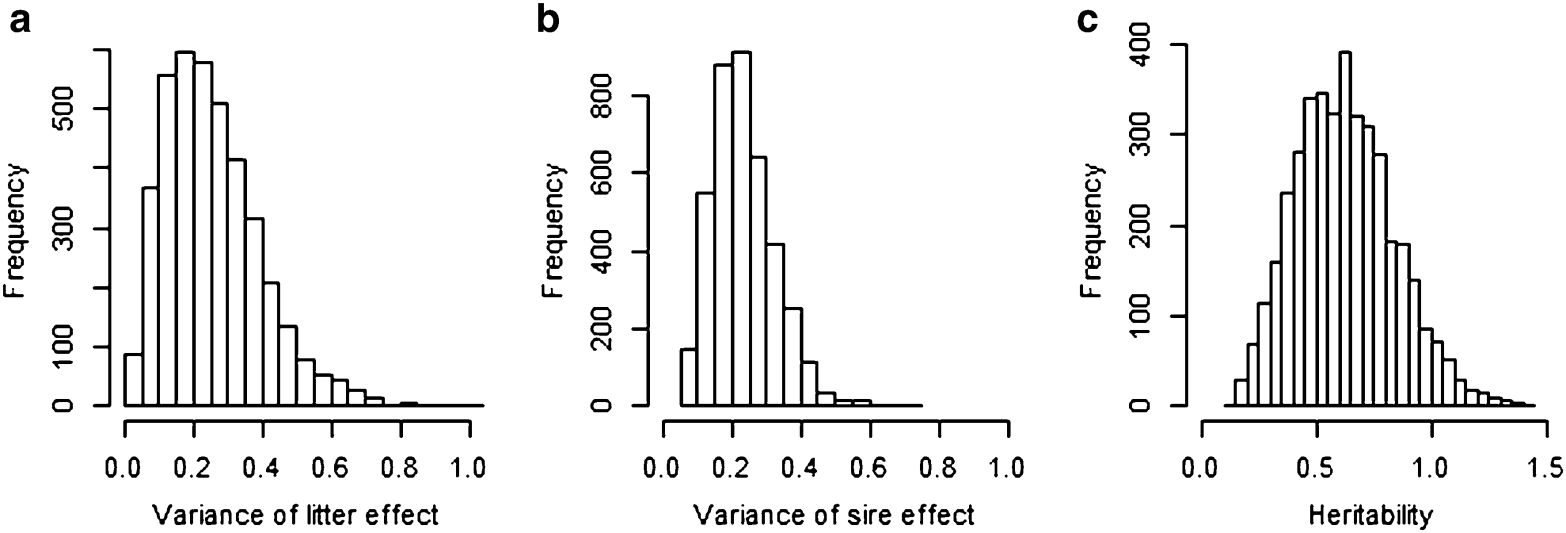
Posterior distribution of the variance of litter (**a**) and sire (**b**) effects and heritability (**c**)

The REML method with the probit model gave an estimated variance of 0.07 (SE = 0.07) for the litter effect and of 0.14 (SE = 0.06) for the sire effect. The estimated heritability of the underlying liability, thus, was 0.47 (SE = 0.18) [Eq. 1]. The smaller estimates for litter effect than for the sire effect is somewhat unexpected because part of the variance related to dams should be accounted by the litter effect. Given the size of the data the REML method may not have been able correctly distribute the underlying phenotypic variance between the sire and litter effects. The marginal posterior densities based on Gibbs sampling for the litter and sire variances and for heritability are presented in Fig. 3. A high value for the mean of the marginal posterior distribution was obtained for heritability (h^2^ = 0.62, SD = 0.21). The corresponding posterior means for the sire and litter variances were 0.23 (SD = 0.09) and 0.26 (SD = 0.14), respectively. The difference in heritability estimates between the two methods (REML and Gibbs sampling) is relatively large but not significant, considering the standard error and standard deviation of the estimates. Also the generalized linear mixed model approach tend to over shrink random effects and their corresponding variances [21]. Thus, the smaller estimates of heritability from the REML method compared to one from Gibbs sampling is expected. Use of a different prior distribution for the variance components (v = 3 or v = 4 instead of v = 6 for the degree of belief of the scaled inverted Chi square distributions) had no effect on the heritability estimates.

The diagnosis of hypothyroidism is challenging, because all affected dogs do not show clinical signs and not all may have been tested in a laboratory. Moreover, notification to the breed club about the disorder is on a voluntary basis, which means that the list of hypothyroid dogs maintained by the Finnish Hovawart Club may be incomplete. Our study data can therefore include dogs given a healthy status although they were, in fact, affected. Also, the data only contained information that is routinely recorded for registration purposes: parents, gender, birth date, color, and breeder, most of which had no influence on susceptibility to hypothyroidism. On the other hand, the data did not enable us to study the effects of other, perhaps more important factors that might increase the risk to acquire hypothyroidism, such as the dogs’ nutritional status or exercise level.

Despite the limitations of the data, our results offer clear evidence that hypothyroidism involves a genetic component. The analysis did not allow to determine whether the genetic component is due to only one or just a few risk genes, or whether the genetic background is more polygenic in nature. Based on the immune-mediated character of hypothyroidism it can be postulated that the variation in the DLA area probably explains at least some of the obtained heritability [5, 12–14]. The high heritability of the disease suggests that a reasonable reduction in its incidence could be obtained by avoiding the use of dogs diagnosed with hypothyroidism and their close relatives in breeding. However, since hypothyroidism can in practice be diagnosed only later in life, it is not possible to exclude all affected dogs from breeding.

## Conclusions

The prevalence of hypothyroidism in the Finnish Hovawart population is relatively high. Our pedigree analysis gave moderate to high heritability estimates for this trait, depending on the method applied. The high heritability of hypothyroidism suggests that selection against it should be effective by using only healthy dogs for breeding.
